# Editorial Commentary: Prophylaxis for Surgical Site Infections

**Published:** 2011-04-01

**Authors:** M Moghadami

**Affiliations:** 1 Health Policy Research Center, Shiraz University of Medical Sciences, Shiraz, Iran

**Keywords:** Prophylaxis, Surgical Site, Infection, Iran

Despite many attempts to prevent surgical site infections (SSIs), these complications are not uncommon in most hospitals while the precise determination of the burden of these infections in our country has not been performed but crude data in hospitals shows a significant cost of managements. With an estimated 27 million surgical procedures annually, and near 2–5% rate of SSIs, approximately 300,000–500,000 surgical site infections can be predicted to occur each year.[1][2][3] In a study in United States, a mean increase of 7-10 days of postoperative hospitalization resulted into higher costs, including increase in annual health care expenditures ranging from 1–10 billion dollars.[4]

Preoperative antibiotics and aseptic techniques are most important aspects of care in major surgical procedures but it should be emphasized that all preventive measures for surgical site infections are unrealistic due to presence of many risk factors that are largely unalterable such as comorbid diseases. So the objectives should be elimination of all potentially preventable infections by the use of evidence based actions.

The center for disease control (CDC) recommends that antibiotic prophylaxis should be used for all clean contaminated procedures and some of clean procedures such as prosthetic joints or intravascular device insertions.[5] Contaminated procedures and dirty wounds often do not need any specific antimicrobial prophylaxis because this type of wound or procedures have already received antibiotic therapy due to presence of an underlying infection. However, if the antibacterial regimen does not adequately cover all microorganisms, additional prophylaxis regimen should be considered. For example, when in specific settings, the risk of methicilin resistant Staphylococoous (MRSA) is highly preventable and the prescribed regimen does not cover MRSA, vancomycin may be recommended.

The benefit of antibiotic prophylaxis in all clean procedures has not been determined. Only in some invasive clean procurers, there are recommendation about antibiotic use and in minimally invasive ones there are few powerful randomized clinical trials and the benefits of their use should be outweighted by potential risks of antibiotic therapy.[6][7]

In this issue of journal, Hatam et al. reported on the economic burden of inappropriate antibiotic use for prophylaxis of infections in surgical wards of Shiraz University of Medical Sciences affiliated hospitals during year 2004. They found that antibiotics were prescribed for 98% procedures in which an antibiotic did not have an indication and also the cost for a 6 months period of antimicrobial prophylaxis regimen was approximately shown 4,623 USD.

The reported study was completed 7 years ago and the research team is to be congratulated on this well designed survey. Those who are working in an infectious ward in university hospitals are painfully aware of inappropriate antibiotic usage in wards including surgical wards and operating rooms. We have a great problem with these unnecessary administrations and need enforcement for reduction of antimicrobial usage.

The inappropriate preoperative use of antibiotics may represent a significant portion of the hospital pharmacy’s expenditure for antibiotics and the surgeons are needed to be encouraged to eliminate antibiotics given for prophylaxis in inappropriate settings. This goal can be reached by educational programs and supervision of administration by infectious disease teams.

However, it seems inappropriate to evaluate antimicrobial use based on cost alone. With inappropriate usage, the over-use or under-use, the costs in terms of mortality, morbidity and resources for fighting against developed resistance microorganism or managing wound infections should be clarified.[8] In these hospitals, the exact cost of prophylaxis therapy with consideration of above mentioned outcome should be determined.

Therefore, one needs to know that such a difficulty exist and cannot be determined simply by direct cost analysis methods. Those whose professional activities are on health economic policy need to design special research projects on all outcomes and direct and indirect costs produced by inappropriate antibiotic usage in hospitals.
